# Introduction of *LPIN1* as a potential diagnostic and prognostic biomarker for gastric cancer via integrative bioinformatics analysis of a competing endogenous RNA network and experimental validation

**DOI:** 10.22038/ijbms.2024.74686.16216

**Published:** 2024

**Authors:** Milad Daneshmand-Parsa, Parvaneh Nikpour

**Affiliations:** 1 Department of Genetics and Molecular Biology, Faculty of Medicine, Isfahan University of Medical Sciences, Isfahan, Iran; 2 Department of Neurochemistry and Psychiatry, University of Gothenburg, Gothenburg, Sweden

**Keywords:** Biomarkers, Competitive endogenous – RNA, Messenger RNA, Pseudogenes, Stomach neoplasms

## Abstract

**Objective(s):**

Identification of effective biomarkers is crucial for the heterogeneous disease of gastric cancer (GC). Recent studies have focused on the role of pseudogenes regulating gene expression through competing endogenous RNA (ceRNA) networks, however, the pseudogene-associated ceRNA networks in GC remain largely unknown. The current study aimed to construct and analyze a three-component ceRNA network in GC and experimentally validate a ceRNA.

**Materials and Methods:**

A comprehensive analysis was conducted on the RNA-seq and miRNA-seq data of The Cancer Genome Atlas (TCGA) stomach adenocarcinoma (STAD) dataset to identify differentially-expressed mRNAs (DEMs), pseudogenes (DEPs), and miRNAs (DEMis). Pseudogene-associated ceRNA and protein-protein interaction (PPI) networks were constructed, and functional enrichment analyses were performed. DEMs and DEPs with degree centralities≥2 were selected for survival analysis. A ceRNA was further selected for experimental validation.

**Results:**

10,145 DEMs, 3576 DEPs, and 66 DEMis were retrieved and a ceRNA network was then constructed by including DEMis with concurrent interactions with at least a DEM and a DEP. Functional enrichment analysis demonstrated that DEMs of the ceRNA network were significantly enriched in cancer-associated pathways. *LPIN1* and *WBP1L* were two mRNAs showing an association with STAD patients overall survival. Expression analysis of *LPIN1* showed a significant decrease in GC tumors compared to non-tumor tissues (*P*=0.003).

**Conclusion:**

Our research emphasizes the significant implications of ceRNA networks in the development of new biomarkers for the detection and prognosis of cancer. Further examination is necessary to explore the functional roles of *LPIN1* in the pathogenesis of GC.

## Introduction

According to the latest GLOBOCAN statistics, stomach or gastric cancer (GC) continues to be a significant global health issue, contributing to more than one million new cases and an estimated 769,000 deaths in 2020 (representing approximately one in every 13 deaths worldwide). It holds the fifth rank in terms of incidence and the fourth rank in mortality globally. The rates of stomach cancer are twice as high in men compared to women. Among men, it is the most frequently diagnosed cancer and the leading cause of cancer-related mortality in various South Central Asian nations, such as Iran, Afghanistan, Turkmenistan, and Kyrgyzstan (1). This disease is diagnosed in advanced and late stages, so its treatment is challenging (2). GC is a multi-factorial and heterogeneous disease, meaning environmental and genetic factors play a role in its formation and development (1, 3). Mechanisms underlying cancers, especially GC, are not fully understood (4). As the need for more efficient methods of diagnosis and treatment of diseases continues to grow, it is crucial to identify new prognostic and diagnostic biomarkers as well as novel therapeutic targets.

In 2011, Salmena *et al*. demonstrated that microRNAs (miRNAs) can mediate a crosstalk among coding and non-coding RNA molecules having shared miRNA response elements (MREs). Different RNA species can be targeted by the same miRNAs and can indirectly calibrate each other by competing for them. These RNAs, also known as competing endogenous RNAs (ceRNAs), lead to the formation of ceRNA networks representing a novel layer of post-transcriptional gene regulation (5). Dysregulation of ceRNA networks has been implicated in different diseases such as cancer. Therefore, studying these networks may lead to a better understanding of cancer pathogenesis, providing novel diagnostic/prognostic biomarkers and developing effective therapeutic strategies for the treatment of cancer (6).

Pseudogenes were initially disregarded due to their lack of transcriptional and protein-coding activities. However, recent research has indicated their involvement in cancer progression, based on duplications, deletion, and additions via retrotransposons (7). Several studies have investigated the relationship between pseudogenes and cancer development (8), including GC (9). For example, the role of phosphatase and tensin homolog pseudogene 1 (*PTENP1*) in the progression of various cancers, has been extensively investigated (10).

Pseudogene, as a particular type of long non-coding RNAs (lncRNAs), can function as RNA sponges for miRNAs, thus exerting a regulatory effect on gene expression through ceRNA networks. However, there are few studies about ceRNA networks including pseudogenes in cancers (11, 12).

**Figure 1:**
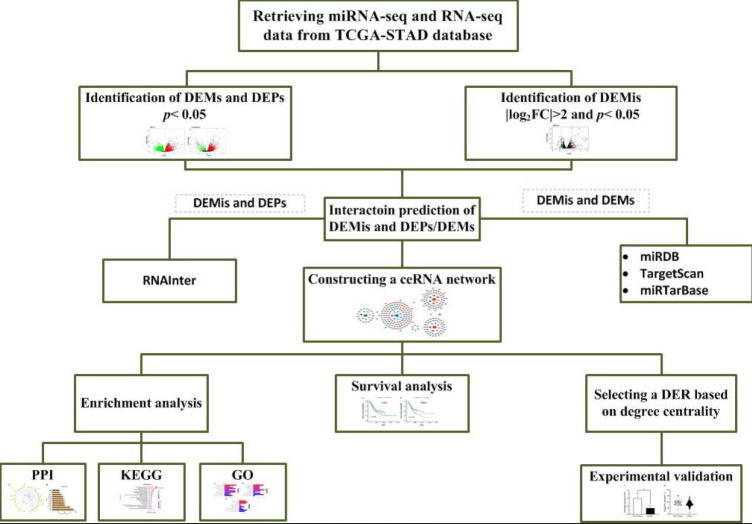
A flowchart of the whole analysis process conducted in the current study

In the present study, we initially conducted a comprehensive analysis of differentially-expressed mRNAs (DEMs), pseudogenes (DEPs), and miRNAs (DEMis) in the stomach adenocarcinoma (STAD) dataset from The Cancer Genome Atlas (TCGA) and constructed a pseudogene-associated ceRNA network for GC. A protein-protein interaction (PPI) network and functional enrichment analyses were then conducted for the mRNAs of the ceRNA network. To experimentally validate the results of the current study, a candidate ceRNA was selected. The selection was based on three-component axes that included different RNA species with the same direction of ceRNA expression changes. The selected axes also had a degree of centralities greater than two. Two axes, “hsa-miR-105-5p, *LPIN1* and *MTCO1P12*” and “hsa-miR-122-5p, *LPIN1* and *MTCO1P12*” met the criteria. As lipin 1 (*LPIN1)*, a phosphatidic acid phosphatase converting phosphatidic acid (PA) to diacylglycerol (DAG), was found to be a survival-related RNA in our analyses, as well, it was chosen for experimental validation in an in-house cohort of GC patients. Dysregulation and association of *LPIN1 *with patients’ survival has been reported in several other cancer types (13-16). Figure 1 presents a flow chart of this study analysis procedure.

## Materials and Methods

***Data retrieval and processing***

The TCGAbiolinks R package was utilized to retrieve RNA- and miRNA-seq data from the STAD-TCGA database, which included 407 (375 tumor and 32 non-tumor) and 491 samples (446 tumor and 45 non-tumor), respectively. The intersected data, which included 372 tumor and 32 non-tumor samples, was normalized utilizing the DESeq2 package. Differential gene expression analysis was performed to identify differentially-expressed RNAs (DERs) with the criteria of |log_2_ fold change (FC)| > 2 and a false discovery rate (FDR) < 0.05 for DEMis. For DEMs and DEPs, a cutoff value of FDR < 0.05 was used.

***Construction of a ceRNA network***

A ceRNA network was constructed including DEMs, DEPs, and DEMis. The miRNA-mRNA interactions were identified utilizing the multiMiR package in the R Studio software with setting the parameters to get the top 20% predictions within each external database. The output was furthermore filtered to include the results of TargetScan predictions (17) with a context++ score of ≤ -0.6, miRDB predictions (18) with a score of > 90, and miRTarBase predictions (19) with the cut-off set to strong evidence. Interactions between DEMis and DEPs were furthermore obtained from the RNAInter online tool (20) with a score of > 0.4. A ceRNA network was then constructed by including DEMis with simultaneous interactions with at least a DEM and a DEP. Cytoscape software (version 3.9.3) was utilized for network visualization, where the genes with the highest degree centrality scores were determined using the cytoHubba plugin (21). The network was made available on the Network Data Exchange (NDEx), a database and online community for sharing and collaborative development of network models (22). 

***PPI network construction***

The PPI network of the DEMs that were included in the ceRNA network was constructed using the Search Tool for the Retrieval of Interacting Genes (STRING) database (23)with a minimum confidence score of 0.4. The resulting PPI network was then imported into the Cytoscape software and likewise made available on the NDEx.

***Functional enrichment analysis***

The ShinyGO 0.77 Bioinformatics tool (24) was utilized to investigate Gene Ontology (GO) and Kyoto Encyclopedia of Genes and Genomes (KEGG) pathway-enriched terms in DEMs of the constructed ceRNA network. All terms with a false discovery rate (FDR) of less than 0.05 were considered statistically significant.

***Survival analysis***

DEMs and DEPs with degree centralities greater than two were chosen from the ceRNA network to analyze the correlation between their expression and TCGA-STAD patients’ overall survival. ‘’survival’’ and ‘’survminer’’ R packages were utilized to determine the association. Samples were divided into two groups based on the median expression of each RNA and the Kaplan-Meier method was used to visualize the results. The thresholds for significance were set at *P*<0.05.

***Clinical specimens***

A total of 60 specimens of GC tumor tissues and adjacent non-tumor tissues were obtained from patients diagnosed with GC. The specimens were collected by Iran National Tumor Bank, which is funded by the Cancer Institute of Tehran University, for cancer research as described previously (25, 26). Prior to their participation, all patients provided written informed consent to the Iran Tumoral Bank. The study protocol was approved by the Ethics Committee of Isfahan University of Medical Sciences (IR.MUI.MED.REC.1400.838) and was conducted in accordance with the Declaration of Helsinki. 

***Total RNA extraction and real-time quantitative reverse transcription PCR (qRT-PCR)***

Total RNA was extracted from both tumor and non-tumor tissues using the QIAzol lysis reagent (Qiagen, Germany), according to the manufacturer’s instructions. RNA purity and concentration were then measured using a Nanodrop spectrophotometer to determine the absorbance of RNA at 230, 260, and 280 nm (Biochrom WPA, UK). The RNA yield was calculated based on the absorbance at 260 nm (A_260_). The ratios A_260_/A_280_ and A_260_/A_230_ were assessed to evaluate RNA purity. For cDNA synthesis, all RNA concentrations were equalized and diluted based on their measurements. In order to eliminate possible genomic DNA contamination, the samples were treated with DNase I (Thermo Scientific, USA). YTA cDNA synthesis Kit (Yekta Tajhiz Azma, Iran) was then used to synthesize cDNA according to the manufacturer’s instructions. Gene Runner (Version 6.3.01 Beta) was used to design specific PCR primers for the *LPIN1* gene (Supplementary Table 1). Basic Local Alignment Search Tool (BLAST) (http://blast.ncbi.nlm.nih.gov/Blast.cgi) was utilized to confirm the primer specificity. A real-time PCR instrument (Biomolecular Systems, Magnetic Induction Cycler (MIC), Australia) was then used to conduct the quantitative RT-PCR assay. The amplification process involved an initial denaturation at 95 °C for 15 min, followed by 40 cycles of denaturation at 95 °C for 20 sec, annealing at 58.5 °C/61 °C for *LPIN1*/*ACTB *(the housekeeping gene) (27) for 30 sec, and an extension step for 30 sec at 72 °C. Real-time PCR was performed with at least three independent technical replicates for each sample. The average measurements from these replicates were utilized for further analysis. Of note, in the case of a high degree of variability in C_t_ of three replicates of a sample, the real-time PCR was repeated for that sample.

***Statistical analysis***

The Livak method (28) was employed to analyze the real-time qRT-PCR data, and GraphPad Prism 9 software was used to conduct statistical analysis. The Kolmogorov-Smirnov test was employed to verify normal statistic distributions of gene expressions. As the data did not follow a normal distribution, the two-tailed Mann-Whitney statistical test, considering *P*<0.05 as statistically significant, was used to compare the mean of gene expressions between tumor and non-tumor gastric tissues. All data were expressed as means ± Standard Error of the Mean (SEM).

Initially, two types of data including expression data and clinical information on TCGA-STAD were downloaded. After determining DEMs, DEPs, and DEMis and their interaction prediction, a network based on DEMis and DEMs/DEPs was constructed and a DER was selected for experimental validation. Moreover, enrichment and survival analyses were conducted on the subnetwork of ceRNA

ceRNA: Competing endogenous RNA, DEMis: Differentially-expressed miRNAs, DEMs: Differentially-expressed mRNAs, DEPs: Differentially-expressed pseudogenes, DER: Differentially-expressed RNA, GO: Gene ontology, KEEG: Kyoto encyclopedia of genes and genomes, PPI: Protein-protein interaction, STAD: Stomach adenocarcinoma, TCGA: The cancer genome atlas

**Figure 2:**
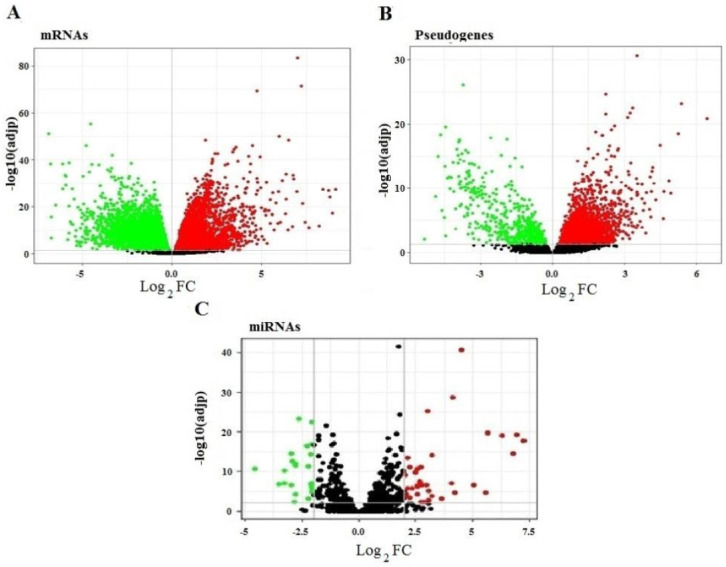
Volcano plots illustrating differentially-expressed RNAs between tumor and non-tumor tissues of TCGA-STAD

Volcano plots of DEMs (A), DEPs (B), and DEMis (C). The horizontal and vertical axes represent -log2 (fold change) and -log10 (adjusted p), respectively. Green and red colors are used to indicate down-regulated and up-regulated RNAs, respectively. The significance threshold for DEMs and DEPs was set as adj. *P*<0.05, whereas the threshold for DEMis was set to adj. *P*<0.05 and |log2 FC|>2.

DEMis: Differentially-expressed miRNAs, DEMs: Differentially-expressed mRNAs, DEPs: Differentially-expressed pseudogenes, FC: Fold change, STAD: Stomach adenocarcinoma, TCGA: The cancer genome atlas

**Figure 3:**
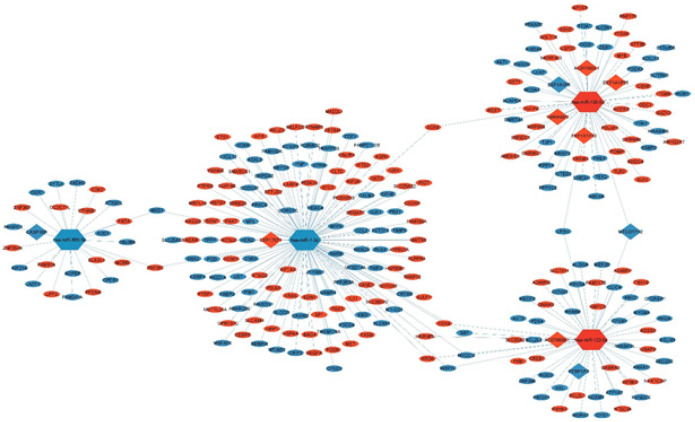
ceRNA network in STAD

Ovals, diamonds, and hexagons represent DEMs, DEPs, and DEMis, respectively. The up-regulated and down-regulated RNAs are represented in red and blue, respectively

ceRNA: Competitive endogenous RNA, DEMis: Differentially-expressed miRNAs, DEMs: Differentially-expressed mRNAs, DEPs: Differentially-expressed pseudogenes, STAD: Stomach adenocarcinoma

**Figure 4:**
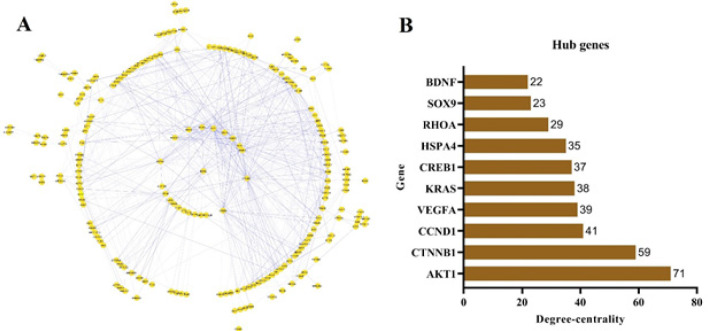
PPI network of the DEMs of the ceRNA network.

A PPI network was constructed showing interactions between 206 nodes and 780 edges. Disconnected nodes were not included in the network (A). A bar plot of the top 10 proteins with the highest degree of centrality was extracted from the PPI network (B).

ceRNA: Competitive endogenous RNA, DEMs: Differentially-expressed mRNAs, PPI: Protein-protein interaction

**Figure 5:**
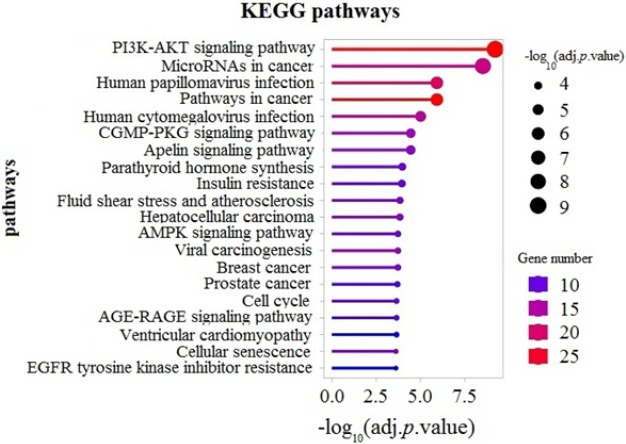
KEGG functional enrichment analysis

The lollipop plot of the most significant 20 KEGG terms. The color indicates the gene number (from the lowest in blue to the highest in red) and the bubble size indicates the -log10 adjusted *P*-value. KEGG terms were ordered based on -log10 adjusted *P*-value (adj.*P*.value)

DEMs: Differentially-expressed mRNAs, KEGG: Kyoto encyclopedia of genes and genomes

**Figure 6:**
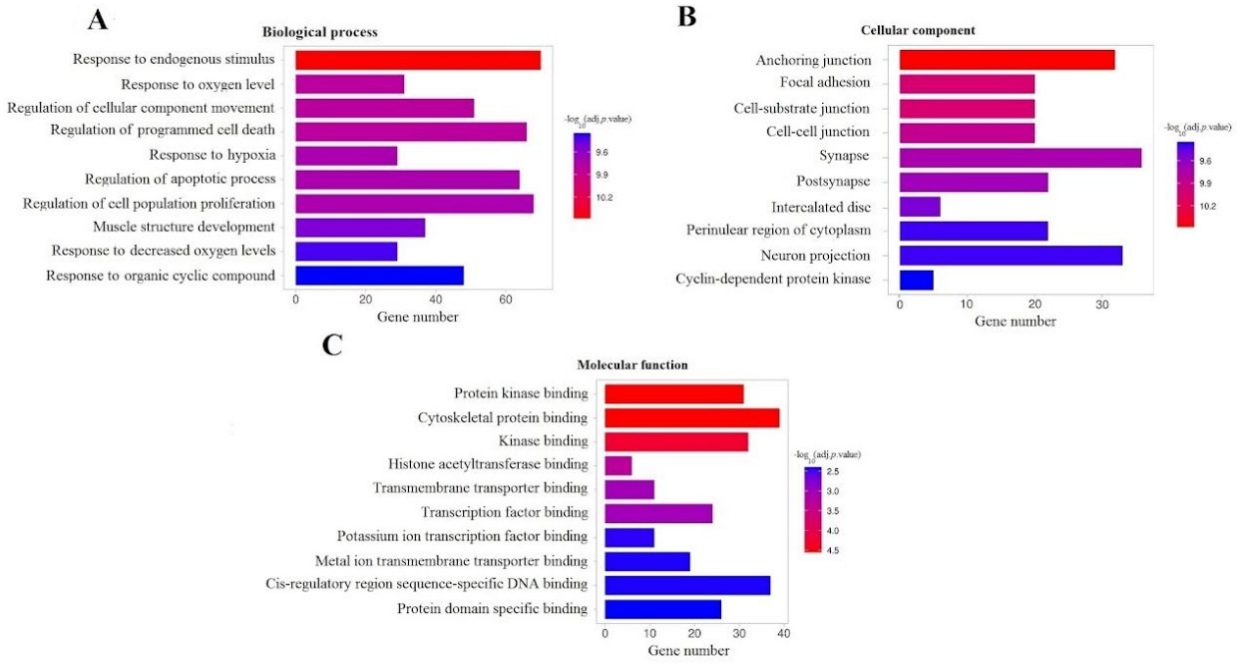
GO analysis on the DEMs of the ceRNA network

The bar plot of the most significant 10 GO terms relating to biological process (A), cellular component (B), and molecular function (C). The horizontal and vertical axes represent gene number and enrichment terms, respectively. GO terms were ordered based on -log10 adjusted *P*-value (adj.*P*.value)

ceRNA: Competitive endogenous RNA, DEMs: Differentially-expressed mRNAs, GO: Gene ontology

**Figure 7:**
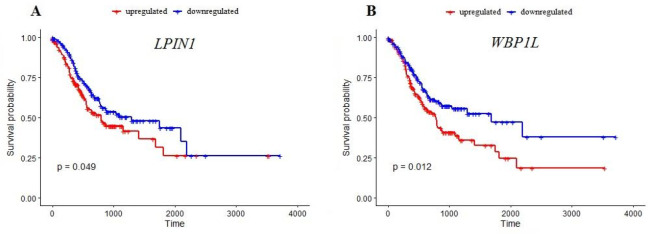
Kaplan–Meier survival curves of the two survival-related RNAs

Survival plots of LPIN1 (A) and WBP1L (B) were constructed by division of TCGA-STAD tumor samples into two groups based on the median of gene expression. Red and blue colors stand for those samples with gene expression higher and lower than the median, respectively

STAD: Stomach adenocarcinoma, TCGA: The cancer genome atlas

**Figure 8:**
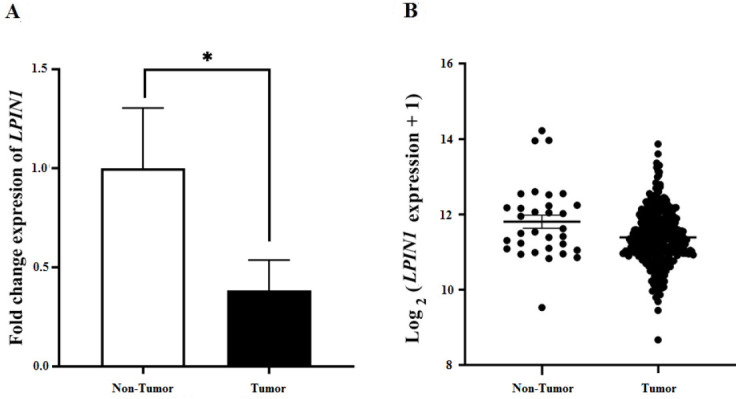
Expression level of LPIN1 in gastric cancer

Relative expression of LPIN1 in an in-house cohort of GC tissue (A) and TCGA-STAD (B) samples which shows reduced expression in tumor vs non-tumor samples with a statistical significance of *P*=0.003 and adjusted *P*-value = 8.69e-5, respectively. Data are presented as mean ± standard error of the mean (SEM).

GC: Gastric cancer, STAD: Stomach adenocarcinoma, TCGA: The cancer genome atlas

**Table 1:** Univariate Cox regression analysis of the correlation of *LPIN1* and *WBP1L* expression levels with overall survival

Gene name	Type	Hazard ratio	*P*	Lower 0.95 CI ^*^	Upper 0.95 CI
*LPIN1*	mRNA	1.39	0.049	0.51	0.99
*WBP1L*	mRNA	1.53	0.012	0.47	0.91

*: Confidence Interval

## Results

***Differentially-expressed RNAs***

The analysis of RNA-seq and miRNA-seq data from the TCGA-STAD database revealed 10145 DEMs (5299 down-regulated and 4846 up-regulated), 3576 DEPs (578 down-regulated and 2998 up-regulated) and 66 DEMis (23 down-regulated and 43 up-regulated) between 372 tumor and 32 non-tumor samples. A visual representation of this data is shown in Figure 2, which displays the volcano plots for each set of DEMs, DEPs, and DEMis. 

***ceRNA network***

To gain insight into the interactions between DEMs and DEMis, the multiMiR package in the R software was employed to access three databases including TargetScan, miRTarBase, and miRDB. Subsequently, the 66 distinct DEMis were used to query the RNAInter database to identify DEPs-DEMis relationships. A ceRNA network containing 277 nodes (263 DEMs, 10 DEPs, and 4 DEMis) and 284 edges was then constructed by including DEMis with concurrent interactions with at least a DEM and a DEP (Figure 3). The network is furthermore available on the NDEx via this link (https://tinyurl.com/yeyn9bfs ).

***PPI network construction and analysis***

A PPI network including 206 nodes and 780 edges (Figure 4A) was constructed to gain a deeper understanding of the interactions between the DEMs of the ceRNA network. AKT1, CTNNB1, CCND1, VEGFA, KRAS, CREB1, HSPA4, RHOA, SOX9, and BDNF (Figure 4B) were the top 10 proteins of the PPI network with the highest degree of centrality. The PPI network is available on the NDEx via this link (https://tinyurl.com/bd38hst9).

***Functional enrichment analyses***

KEGG pathway analysis was performed utilizing the ShinyGO tool. Figure 5 shows the top 20 enriched pathways with the lowest adjusted *P*-values. This analysis revealed that the ceRNA network DEMs were enriched in cancer-related pathways such as the “PI3K-Akt signaling pathway”, “microRNAs in cancer” and “Pathways in cancer”. Plots of the most significant ten GO terms relating to biological processes, cellular components, and molecular function are depicted in Figure 6.

***Survival analysis of DEMs and DEPs of the ceRNA network***

372 TCGA-STAD samples containing clinical information were divided into two groups, low expression and high expression, based on the median expression of each DEM and DEP of the constructed ceRNA network which had degree centralities greater than two. Based on the univariate Cox regression, it was determined that the overall survival of patients was significantly associated with 2 of the 11 RNAs (*LPIN1* and *WBP1L*, with *P*=0.049 and *P*=0.012, respectively). Figure 7 displays the Kaplan-Meier plots for the two RNAs. Furthermore, the hazard ratios (HRs) and confidence intervals (CIs) of these two RNAs are summarized in Table 1.

***Relative quantification of LPIN1 expression in gastric tissues***

To select a candidate ceRNA to experimentally validate the results of the current study, we focused on three-component axes which included all three different RNA species, showing the same direction of ceRNA expression changes that should be opposite of the miRNA expression changes (according to the ceRNA hypothesis) as well as having degree of centralities greater than two. Two axes including “hsa-miR-105-5p, *LPIN1* and *MTCO1P12*” and “hsa-miR-122-5p, *LPIN1* and *MTCO1P12*” met the criteria and as *LPIN1 *was found to be a survival-related RNA as well, it was selected for experimental validation. The RNA levels of *LPIN1* were found to be significantly (*P*=0.003) decreased in GC tumors compared to non-tumor tissues. The same trend was furthermore observed in the TCGA-STAD samples (Figure 8).

study has comprehensively analyzed a three-component mRNA-pseudogene-miRNA ceRNA network in GC. For instance, in 2022, a differential-expression analysis was initially performed on five GC Gene Expression Omnibus (GEO) datasets, and then miRNAs and pseudogenes/lncRNAs that might combine with *COL5A2*, a hub DEM, were identified and its ceRNA network was constructed (31). The main difference between this study and ours is that they mainly focused on a ceRNA axis and did not construct a general ceRNA network via a hypothesis-free approach.

Analysis of the PPI network showed that the top 10 proteins with the highest degree of centrality all have associations with neoplasms and cancer. Then, an enrichment analysis was conducted on the DEMs of the ceRNA network, which revealed the most enriched signaling pathways and GO terms that were cancer-related like “PI3K-Akt signaling pathway”, “microRNAs in cancer”, and “Pathways in cancer”. Pieces of Evidence show that the PI3K–Akt signaling pathway is one of the most pivotal intracellular pathways, which regulates survival, cell growth, differentiation, cellular metabolism, and cytoskeletal reorganization of cells. Deregulation of this pathway has been documented in several cancer types (32).

Survival analysis showed that the overall survival of TCGA-STAD patients was significantly associated with expression of *LPIN1* and *WBP1L*. Higher expression of *WBP1L*, which is also referred to as outcome predictor in acute leukemia 1 (*OPAL1*) was firstly reported to be associated with favorable prognosis in patients with acute lymphoblastic leukemia (ALL) (33), however, a subsequent study reported that OPAL1 expression may not be an independent prognostic feature in childhood ALL (34). *LPIN1* (lipin 1) is a protein-coding gene that is primarily involved in lipid metabolism. Dysregulation of fatty acid metabolism is generally recognized as a player in malignant transformation in different cancer types (35, 36). Recent pieces of evidence show that *LPIN1* plays a critical role in the regulation of the PI3K-AKT-mTOR pathway, a common dysregulated pathway in most cancers (16). Association of *LPIN1 *with prognosis has been furthermore reported in several cancer types including breast cancer (37), lung adenocarcinoma (38), and head and neck squamous cell carcinoma (39).

As a survival-related gene, appearing in two axes in the constructed ceRNA network and showing a gene expression pattern compatible with the ceRNA hypothesis (5), *LPIN1* was selected as a candidate gene for experimental validation in the current study. Consistent with its gene expression pattern in TCGA-STAD patients, we also observed its lower expression in GC tumors compared to non-tumor tissues. Dysregulation of *LPIN1* gene expression has been previously documented in several cancer types like breast cancer (40), lung adenocarcinoma (15), and prostate cancer (14). 

## Conclusion

we conducted a comprehensive analysis of gene expression profiles in GC patients from TCGA and presented a three-component ceRNA network consisting of three types of RNAs including mRNAs, pseudogenes, and miRNAs. *LPIN1* expression was shown to be associated with the overall survival of patients with stomach adenocarcinoma and its dysregulation was experimentally confirmed in a cohort of GC tissues. Our study highlights the important implications of ceRNA networks to introduce novel biomarkers for cancer diagnosis and prognosis. Further studies investigating the functional roles of *LPIN1* in GC pathogenesis are highly demanded.

## Data Availability

The results published or shown here are in whole or part based upon data generated by the TCGA Research Network: https://www.cancer.gov/tcga .

## Fuding Statement

This work was partially supported by a grant from Isfahan University of Medical Sciences, Isfahan, Iran. The sponsor (Isfahan University of Medical Sciences) had no role in the study design, nor in collection, analysis, or interpretation of data; in the writing of the manuscript; or in the decision to submit the manuscript for publication.

## Appendix

**Table S1:** Specific PCR primers for *LPIN1* and *ACTB*

**Transcript**	**Sequence (5' to 3')**	**Product size**
***ACTB***	**F:** TTCGAGCAAGAGATGGCC	151 bp
	**R:** CACAGGACTCCATGCCCAG	
***LPIN1***	**F:** AAAGGACAGGGCAGAAGAAC	176 bp
	**R:** AGTGTCTGAAGATTCGCTGTG	
